# Hypertension in malignancy–an underappreciated problem

**DOI:** 10.18632/oncotarget.25024

**Published:** 2018-04-17

**Authors:** Jolanta Małyszko, Maciej Małyszko, Leszek Kozlowski, Klaudia Kozlowska, Jacek Małyszko

**Affiliations:** ^1^ 2nd Department of Nephrology and Hypertension with Dialysis Unit, Medical University in Bialystok, Bialystok, Poland; ^2^ Department of Nephrology, Dialysis and Internal Medicine, Warsaw Medical University, Warsaw, Poland; ^3^ Department of Oncological Surgery, Regional Cancer Center, Bialystok, Poland; ^4^ 1st Department of Nephrology and Transplantology with Dialysis Unit, Medical University in Bialystok, Bialystok, Poland

**Keywords:** malignancy, hypertension

## Abstract

Hypertension is one of the most common comorbidities in cancer patients with malignancy, in particular, in the elderly. On the other hand, hypertension is a long-term consequence of antineoplastic treatment, including both chemotherapy and targeted agents. Several chemotherapeutics and targeted drugs may be responsible for development or worsening of the hypertension. The most common side effect of anti-VEGF (vascular endothelial growth factor) treatment is hypertension. However, pathogenesis of hypertension in patients receiving this therapy appears to be associated with multiple pathways and is not yet fully understood. Development of hypertension was associated with improved antitumor efficacy in patients treated with anti-antiangiogenic drugs in some but not in all studies. Drugs used commonly as adjuvants such as steroids, erythropoietin stimulating agents etc, may also cause rise in blood pressure or exacerbate preexisiting hypertension. Hypotensive therapy is crucial to manage hypertension during certain antineoplastic treatment. The choice and dose of antihypertensive drugs depend upon the presence of organ dysfunction, comorbidities, and/or adverse effects. In addition, severity of the hypertension and the urgency of blood pressure control should also be taken into consideration. As there are no specific guidelines on the hypertension treatment in cancer patients we should follow the available guidelines to obtain the best possible outcomes and pay the attention to the individualization of the therapy according to the actual situation.

## INTRODUCTION

With the recent success of modern cancer therapy, cancer can be curable, and in cases where cure cannot be achieved, it can be treated as a chronic disease. As a result, there are now more than 13 million cancer survivors in the US alone [1] and close to 30 million worldwide [2]. Given the growing population of patients once treated (or continuing treatment) for cancer, the medical community must learn how to best minimize the complications of cancer treatment.

The effects of anticancer treatment on cardiovascular system are of utmost importance to the overall well-being of cancer survivors [3] as a growing number of subjects with higher prevalence of hypertension, valvular disease, cardiomyopathy and heart failure, and pulmonary disease compared with the general population [4]. Due to the fact that, these comorbidities are associated with higher morbidity and mortality, the most important priority is the recognition of the necessity of improvement prevention, diagnosis, and therapy of cardiovascular and pulmonary disorders.

## HYPERTENSION AS A COMORBIDITY

Hypertension is a long-term consequence of many cancer therapies, including both chemotherapy and targeted agents. Arterial hypertension appears to be the most common entity in cancer patients and its incidence is rising in line with growing population of the elderly in the developed world [5]. It has been reported that prevalence of hypertension in patients with malignancy was around 30% [6]. It has been also the most common comorbidity reported in cancer registries [7]. However, the detailed data are lacking.

## HYPERTENSION AS A COMPLICATION OF THE THERAPY

Some cancers such as renal cell carcinoma may cause secondary hypertension. What is even more important that some active treatments i.e. inhibitors of vascular endothelial growth factor-VEGF receptor may lead to or worse previously well controlled hypertension [8–12]. It has been reported that incidence of overall hypertension was 20–44% and the high-grade hypertension was 6–17%, in particular during active therapy [8–12]. Prevalence of hypertension depends upon age, prior hypertension or cardiovascular disease in anamnesis, type of malignancy (renal or non-renal), type of therapy and dose, chemotherapy regimen and concomitant medications. However, the long-term effects on blood pressure are unknown. Table 1 present antineoplastic agents, type of nephrotoxicity, mechanisms, renal adverse effects preventive measures and proposed hypotensive drugs.

**Table 1:** Anticancer drugs, type of nephrotoxicity, mechanism and prevention of renal adverse events

Medication	Cardiotoxicity	Mechanism of action	Likelihood of HT	Proposed hypotensive therapy
Alkylating agents cyclophosphamide	HT	endothelial dysfunction, arterial vasoconstriction, renal and vascular damage	+	RAAS blockade (ACEi, ARB)
Antimetabolites methotrexate gemcitabine	HF, HT,	Drug-induced- thrombotic microangiopathy-DITMA	+	
mTOR	HT	Podocyte damage,	+	RAAS (ACEi, ARB)
Platinum derivatives	HT	Oxidative stress, renal damage	+	
Proteasome inhibitors	Drug-indiced thrombotic microangiopathy		+	
Anti-angiogenesis drugs VEGF pathway inhibitors- Bevacizumab, Aflibercept Sorafenib Sunitinib Pazopanib Vandetanib Axitinib Regorafenib cabozantinib	hypertension thrombotic microangiopathy	Peripheral vascular resistance, reduced formation of nitric oxide in endothelium, increased synthesis of vasoconstrictive factors, kidney damage	+++	RAAS (ACEi, ARB) CCB
glucocorticosteroids	HT	Salt and volume overload	+	diuretics
anthracyclines	LVD, HF/HT	Oxidative stress, apoptotic/fibrotic changes in vascular wall, endothelial dysfunction	+	RAAS (ACEi, ARB), beta-blockers
HER2 inhibitors	LVD, HF/HT	Oxidative stress, apoptotic/fibrotic changes in vascular wall, endothelial dysfunction	+	RAAS (ACEi, ARB), beta-blockers

Anticancer drugs, type of nephrotoxicity, mechanism and prevention of renal adverse events.

HT-hypertension, HF- heart failure, LVD- left ventricular dysfunction, mTOR- mammalian target of rapamycin, VEGF- vascular endothelial growth factor, RAAS-renin-angiotensin-aldosteron system, ACEi – angiotensin converting enzyme inhibitor, ARB- angiotensin receptor blocker, CCB- calcium channel blockers.

### Cisplatin derivatives

Among chemotherapeutic agents, the most data have been reported for cisplatin and come from basic science data and from the evaluation of men treated for testicular cancer [13, 14]. Unfortunately, few data exist for women who received a platinum agents. Cisplatin exerts cytotoxic effects via the formation of covalent adducts with DNA purine bases and inter and intra-strand cross-links, which can persist in multiple organ systems and circulate for many years after exposure [15–17]. At the vascular level, cisplatin appears to abolish capillary beds [18]. Furthermore, animals treated with cisplatin demonstrate increased levels of tumor necrosis factor (TNF) alpha and multiple cytokines [19] and markers of oxidative stress [20]. Although speculative, these factors may help to explain long-standing toxicities related to cisplatin, including hypertension. In survivors of testicular cancer observed for 11.2 years (median), has a higher blood pressure level together with a risk of incident hypertension, which was significantly elevated relative to healthy controls (odds ratio [OR] 1.4, 95% CI 1.2–1.7). [13]. Subgroup analyses demonstrated that the age adjusted odds of hypertension was greatest in the group given cisplatin, particularly at dosages >850 mg (OR 2.4, 95% CI 1.4–4.0). Another study showed that testicular cancer survivors, who had received chemotherapy and were observed for 19 years (median), had an increased prevalence of antihypertensive medication use compared with the general population (OR 3.7, 95% CI 1.9–5.2) [14].

### Proteasome inhibitors

Chari and Hajje [21] reported the retrospective data on 67 myeloma patients with patients with relapsed and/or refractory disease treated at Mount Sinai Medical Center, USA. They described 12 patients who suffered from either or cardiac or vascular-related side effects associated with carfilzomib-based treatment (median age was 59 years, with ranges from 49 to 77 years). In one case the hypertension and lack of other signs of renal impairment suggested that the side effect was of vascular origin. In another subject, acute exacerbation of chronic hypertension during carfilzomib treatment was probably due to renal fibromuscular dysplasia. The authors recommended to carefully evaluate the blood pressure and hypotensive treatment, in order to diminish the risk of kidney impairment. In patients with chronic kidney disease, it is of utmost importance to establish if whether the worsening of kidney function is due to the therapy or to progression of the diseases to introduce the appropriate management of this condition.

### Anthracyclines

It has been known that anthracyclines are responsible for congestive heart failure, especially when given in high cumulative doses [22]. However, in 1979 von Hoff *et al.* [23] retrospectively analyzed 4018 patients from the cooperative group trials and described for the first time the association between doxorubin toxicity and hypertension. Hypertension was a predisposing factor for development of congestive heart failure. Similar data were published by Hequet *et al.* [24] who found that preexisting hypertension was a risk factor for late subclinical cardiomyopathy in subjects with lymphoma treated with anthracyclines as well as in breast cancer patients [25]. In 9,438 subjects with DLBCL- diffuse large B-cell lymphoma, 3,164 (42%) received doxorubicin-based chemotherapy, 73% of them had hypertension, hypertension was synergistic with doxorubicin to cause development of chronic heart failure [26]. The possible mechanism is multifactorial and include oxidative stress with apoptotic/fibrotic inflammatory changes in vascular wall together with endothelial dysfunction [25–28]. Heart failure is the major complication after anthracyclines given with or without trastuzumab. As shown by Russo *et al.* [29] new onset chronic heart failure with a significant reduction in left ventricular ejection fraction was predicted by a history of hypertension. In addition, cardiotoxicity caused by breast cancer therapy was increased in smokers, patients with obesity, dyslipidemia, diabetes, hypertension or prior history of cardiovascular disorders. Moreover, randomized controlled trials did report consistently decreased cardiotoxicity than found in observational studies [30]. Therefore, diagnosis of hypertension (using new American Heart Association-AHA guidelines from 2017) [31] and timely and appropriate treatment may diminish the incidence of heart failure related to cancer therapy.

### Gemcitabine

Gemcitabine, is a pyrimidine antagonist, that was linked with thrombotic microangiopathy-TMA [32] Recently, it has been reported that 29 patients gemcitabine-associated TMA also developed acute kidney injury-AKI. Hypertension, either de novo or worsening of the preexisting was found in 26 subjects, while congestive heart failure was observed in 7 cases. Withdrawal of the offending causative drug is the primary approach for TMA associated with chemotherapy. Improved clinical performance is seen after withdrawal in some, but not all instances [33, 34].

### Mammalian target of rapamycin-mTOR inhibitors

Inhibitors of mTOR such as everolimus, temsirolimus, and ridaforolimus have shown anticancer activity in various malignances, most notably advanced renal cell carcinoma-RCC [35–37]. However, some their immunosuppressive and anticancer properties are linked with several side effects such as diabetes, hyperlipidemia, proteinuria, or hypertension) [38–40] and others [41].

### Other drugs

Alkylating agent cyclophosphamide has been reported to be associated with cardiotoxicity hypertension probably by causing endothelial dysfunction, arterial vasoconstriction together with renal and vascular damage [42, 43].

Glucocorticosteroids, mainly dexamethasone, are used commonly as adjuvants and may cause hypertension due to salt and volume retention [44, 45]. Erythropoietin stimulating agents used also as adjuvant to treat chemotherapy-induced anemia may be prohypertensvive as they increase erythrocyte mass and blood viscosity and direct vasopressor effect [46–48].

## VEGFR AND HYPERTENSION

VEGF is crucial in vascular homeostasis. It mediates the synthesis of the vasodilator nitric oxide, and generation of new blood vessel leading to decreased vascular resistance [49–53]. This role of VEGF is associated with decline in blood pressure. Therefore, inhibition of VEGF signaling could lead to development or worsening of preexisting hypertension [54, 55]. VEGF signaling inhibitor induced elevation in blood pressure appears to be not an adverse event of the therapy, but rather a mechanism-dependent on-target toxicity [56]. Taking these data into consideration, all trials evaluating inhibitors of angiogenesis have restricted eligibility to patients with controlled blood pressure at baseline. All commercially available angiogenesis inhibitors have been implicated in the development of hypertension, including bevacizumab [57–60], aflibercept [61], sorafenib [62], sunitinib [63, 64], pazopanib [65], vandetanib [66], axitinib [62, 67], regorafenib [68], and cabozantinib [69, 70].

**Figure 1:**
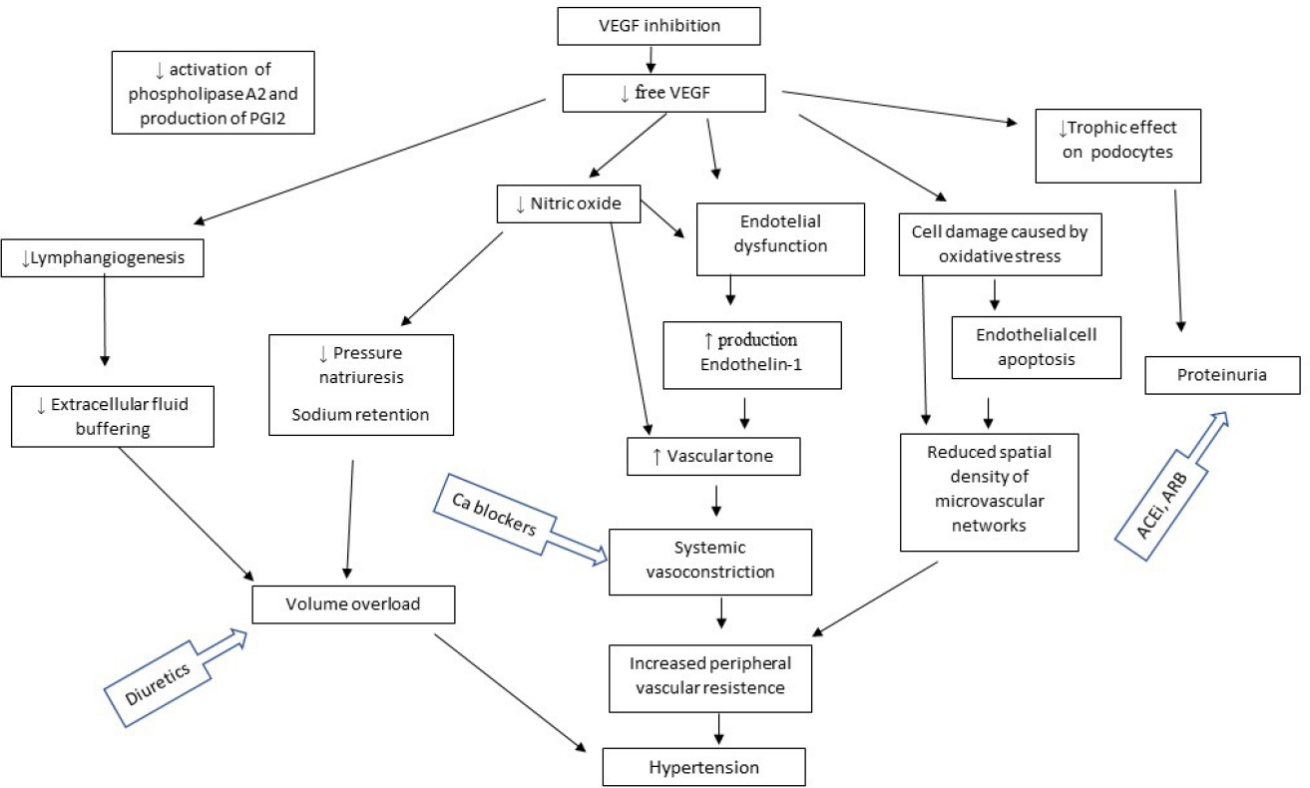
Proposed mechanisms of hypertension induced by anti-VEGF therapy (modified from 128)

However, the pathogenesis of elevated blood pressure in subjects treated with anti-VEGF drugs appears to be associated with multiple pathways so far is not yet fully elucidated. The proposed mechanism is shown on the Figure 1. The pathophysiological mechanism of hypertension induced by anti-angiogenic therapy include increased peripheral vascular resistance together with diminished nitric oxide synthesis in endothelium, an increased synthesis of vasoconstrictive substances, and a decreased density in microvasculature i,e rarefaction. In addition, impaired renal function may also contribute to the development or worsening of hypertension. The mechanism of worsening of kidney function during VEGF-TKI (tyrosine kinase inhibitors) therapy has not been elucidated in details. Inhibition of VEGF by pharmacotherapy results in glomerular, endothelial and podocyte injury. It may lead to proteinuria as well. In kidney biopsy, the most common were TMA, reflecting vascular damage [71], followed by glomerulonephritis with crescents, focal segmental glomerulosclerosis (FSGS), glomerulonephritis with immune complexes, minimal change disease (MCN), and acute interstitial nephritis [72–76]. In a case of nephropathy, in particular, with impaired kidney function and volume overload, hypertension is common. In addition, therapy with anti-VEGF drugs causes vasoconstriction due to diminished synthesis of NO and prostacyclin-PGI2, which leads to impaired blood flow in the glomeruli. It should be also stressed that worsening of kidney function may result from the nephrectomy in RCC subjects as the nephrons loss during nephrectomy either partial or radical is a predisposing factor for chronic kidney disease or development of contrast-induced nephropathy following computed tomography (CT) with contrast media in any malignancy.

### Incidence and characteristics

The study of Miyake *et al.* [77] reported that hypertension development during sunitinib-therapy predicted good tumor response and significantly longer progression free survival-PFS, but not overall survival-OS, in metastatic RCC patients. Furthermore, the incidence of hypertension induced by sunitinib treatment was linked to a longer progression-free survival. Their data are similar to the published reports on hypertension-induced by bevacizumab and increased PFS [78] and between hypertension induced by axitinib with increased OS [79]. Sire *et al.* [80] reported that hypertension (grade2–4) developed during first- or second line sunitinib, sorafenib, or bevacizumab therapy for metastatic RCC was a favorable prognostic factor. Moreover, some toxicities caused by sunitinib, such as hypertension and hypothyroidism, as predictors should be tested and validated in well-designed prospective randomized controlled trials.

Bondarenko *et al.* [81] studied the efficacy and safety of combined therapy including axitinib with cisplatin/ gemcitabine in chemotherapy-naïve subjects with squamous non– small-cell lung cancer (NSCLC) in advanced/metastatic settings (stage IIIB/IV). They reported that most neutropenia and hypertension (13.2% each) were the most common grade ≥ 3 toxicities while hypertension was found in 26.3%, (they excluded patients with uncontrolled hypertension above 140/90 mm Hg). Hypertension as the most common cause for reduction of the dose of axitinib. Anlotinib, a novel multi-target TKI developed to primarily inhibit VEGFR2/3, fibroblast growth factor receptor-FGFR1–4, platelet-derived growth factor receptor-PDGFR α/β, c-Kit, and Ret. Sun *et al.* [82] evaluated the anlotinib safety, pharmacokinetics, and antitumor activity in advanced/refractory solid tumors. They reported that hypertension was the main serious adverse effect. Sulfatinib (HMPL012) is a potent small molecule TKI of VEGFR 1, 2, and 3, FGFR 1, and Colony Stimulating Factor 1 Receptor- CSF1R. Xu *et al.* [83] investigated the sulfatinib safety, pharmacokinetics, and preliminary antitumor activity in advanced solid tumors. As for other anti-angiogenic drug the most frequent side effects during therapy were proteinuria, hypertension and diarrhea.

Mittal *et al.* [84] studied a combination of bevacizumab- VEGF antibody, and sunitinib- inhibitor of VEGFR, in patients with advanced solid tumors. Grade 3 or higher side effects, such as hypertension was observed in 41%. In addition, in 18% TMA was reported. Development of TMA associated with dual VEGF/VEGFR inhibition could be due to systemic or kidney injury even in malignances of non-renal origin. Similarly, when sorafenib was combined with bevacizumab in heavily pretreated subjects with advanced solid tumors, this therapy was associated with hypertension and hand-foot syndrome. However, development of hypertension (grade 3 and 4) was associated with longer time to therapy failure, OS, and higher response rate. In subjects with metastatic melanoma with the ECOG- Eastern Cooperative Oncology Group performance status 0–1 and normal organ function in a prospective phase II trial therapy with axitinib followed by paclitaxel/carboplatin resulted in development of hypertension in 41%, grade 3 hypertension was observed in 16%. [85]. In the study of Tomita *et al.* [86] axitinib was administered to Japanese (n=) 44 and non-Japanese (n=169) patients with metastatic RCC, which were treatment-naïve. The most frequent side effects in both populations were hypertension and diarrhea. However, most common adverse events, such as hypertension and proteinuria, were more prevalent in Japanese subjects, who also received more often hypotensive medications (95% in Japanese patients vs. 64% in non-Japanese patients). In patients with RCC, treatment with pazopanib and sunitinib resulted in a similar incidence of hypertension (46% vs 41%), however axitinib caused more hypertension more than sorafenib (40 vs 29%) [62, 87] Ramucirumab was responsible for 8% of cases of severe hypertension in patients with advanced gastric cancer [88]. Aflibercept given to patients with colorectal cancer was associated with severe hypertension in 19.1% of cases [61]. It appears that levantinib was associated with the significantly high rate of both hypertension (68%) and severe hypertension (42%) [89]. An *et al.* [90] published a meta-analysis, which included 12,949 patients with advanced solid tumors treated with or without bevacizumab. They found that the relative risk (RR) of development or worsening of hypertension (defined as more than 1 hypotensive drug used, or for a more intensive therapy than previously, or life-threatening complications such as hypertensive crisis; grade 3 or 4 among patients receiving bevacizumab was 5.38 (95% CI 3.63–7.97) and was dose-dependent [90]. Moreover, overall incidence of hypertension in bevacizumab-treated patients was 24% (95% CI 20–29 percent), whereas significant rise in blood pressure was found in 8% (95% CI 6–10 percent). Zhu *et al.* [91] in another meta-analysis analyzed the incidence of hypertension in 13 prospective studies with 4999 subjects with RCC or other malignancies administered sunitinib. The incidence of hypertension was 22%, while in 7% hypertension was described as severe. Sunitinib treatment was a significant risk factor for development of severe hypertension (RR 22.72, 95% CI 4.48–115.29). Similar results yielded a systematic review of 9 prospective studies including 4599 patients treated with sorafenib [92]. Another meta-analysis, which included 18 randomized Phase II and III controlled trials on subjects with solid tumors treated with sorafenib reported that daily sorafenib was responsible for enhanced risk for development of hypertension (all grades) and bleeding relative to the control group [93]. In one more meta-analysis by the same authors, it has been has demonstrated that sunitinib, axitinib, cediranib and regorafenib were also the risk factors for development of hypertension (both all grades and high grade) relative to the control group [94]. On the other hand, in one meta-analysis, pazopanib therapy results in higher incidence of hypertension than sorafenib or sunitinib (36 versus 23 and 22%, respectively), but similar incidence of severe one (6.5 versus 5.7 and 6.8%, respectively) [95]. In the other meta-analysis, sorafenib, sunitinib, and trastuzumab, were associated with increased risk for reduced left ventricular ejection fraction and hypertension [96, 97]. In the recent review Kroschinsky *et al.* [98] stressed that introduction of angiogenetic pathways inhibitors substantially enlarge the armamentarium of targeted therapies. As angiogenesis is also necessary for repair of tissue and regeneration thus, effect of these therapies on vascular system, such as severe adverse effects in a form of thromboembolism, bleeding from gastrointestinal tract or even perforation, de novo or worsening of hypertension, and development of congestive heart failure, compromise antineoplastic efficacy. In the recent systemic review, Semeniuk-Wojtas *et al.* [99], included 48 eligible phase III and IV prospective clinical trials, meta-analyses and retrospective studies describing the AEs in a form of hypertension or other nephrotoxicity in patients received anti-VEGF drugs. They found that hypertension (any grade) was reported in 17% - 49.6% of patients, while proteinuria and elevated serum creatinine were found in 8% to 73% and 5% to 65.6% of subjects, respectively. Risk factors for rise in blood pressure under VEGFR therapy are prior hypertension, age ≥ 60 years, and body mass index (BMI) ≥ 25 kg/m^2^ [100]. The significant rise in blood pressure were observed as early as in the first week of treatment [101, 102]. There are several reports that the are some SNPs that are linked with a higher risk for hypertension development during TKI therapy [103, 104]. However, no factors are yet known to predict the magnitude of blood pressure rise [105]. As untreated severe hypertension may lead to serious complications, patients treated with VEGFR inhibitors should have their blood pressure actively monitored during therapy, with more frequent measurement in the first several weeks of the treatment.

### Association with antitumor efficacy

It has been reported that hypertension development was linked with better antitumor efficacy in subjects treated with bevacizumab and the antiangiogenic TKIs [58, 104, 106–112] however, it was not a consistent finding in some studies [113–115]. In 4 prospective studies in patients with advanced RCC treated with sunitinib, rise in blood pressure over 140 mm Hg was associated with better antitumor efficacy (median OS, median PFS, and objective response rates were 30.9 versus 7.2 months, 12.5 versus 2.5 months, and 55 versus 9 percent, respectively) [111]. Similar results were seen with rise in diastolic blood pressure over 90 mmHg. Moreover, multivariate analysis revealed that sunitinib treatment independently predicted survival (hazard ratio [HR] 0.28, 95% CI 0.22–0.37). Six clinical studies of sunitinib, sunitinib showed that dose intensity and cumulative weekly dose correlated with both improved clinical outcomes and maximum blood pressure [116]. On the other hand, in 7 randomized trials with bevacizumab used for RCC, colorectal, breast, NSCLC, and pancreatic cancer, no association between early hypertension and clinical benefit from bevacizumab was found [115].

Hence, further studies are warranted to validate hypertension as an efficacy predictor during therapy with angiogenesis inhibitors. It has been reported that hypertension development predicted a better response of the tumor to the therapy with anti-VEGF drugs in some studies [32, 117, 118], therefore, in a case of either hypertension de novo or worsening of the preexisting hypertension, physicians should maintain targeted therapy and use of hypotensive medications to control blood pressure rather than withdraw antineoplastic drugs. However, withdrawal of anti-VEGF treatment should be taken into account in cases when severe adverse events appeared. It should also stressed that treatment with bevacizumab combined with TKIs may result in development of severe hypertension and other life-threatening toxicities i.e. vascular and hematological [119, 120].

### Monitoring and management of hypertension

For most studies and in clinical practice, patients who are actively taking antihypertensive medications are usually defined as having hypertension regardless of their observed blood pressure. There are no data in this aspect. Ambulatory blood pressure monitoring is generally employed in the randomized control trials in hypertension, however, not in every study i.e. SPRINT (Systolic Blood Pressure Intervention Trial, ClinicalTrials.gov identifier NCT01206062) where blood pressure was measured by patients in office in the seated position using an automated measurement device (Omron Healthcare, Lake Forest, IL) [121] In some cases of incurable disease, a minimalistic approach to treat elevated blood pressure might be favored, however, treatment of existing comorbidities, with hypertension among them, actually may improve quality of life and survival [116]. Furthermore, appropriate control of blood pressure should allow oncology subjects to cope with the highest effective doses of the treatment, for longest period of time [102]. Patients who develop hypertension during treatment (by definition as blood pressure ≥140/90 mmHg or a 20 mm increase in diastolic blood pressure over baseline) should be treated with antihypertensives. Target blood pressure is below 140/90 mm Hg or even lower in case of over proteinuria according to current guidelines (ESH/ESC-European Society of Hypertension/European Society of Cardiology, AHA-American Heart Association, JNC8-Joint National Committee) [122–124]. However, according to new AHA guidelines hypertension stage 1 is diagnosed when systolic blood pressure is between 130 to 139 mmHg or diastolic blood pressure is between 80 to 89 mmHg, stage 2 is defined when systolic blood pressure is at least 140 mmHg or diastolic blood pressure is at least 90 mmHg on 2 or more properly measured readings at each of 2 or more office visits after an initial screening [31]. Organ dysfunction and/or adverse effects on organ function may influence the choice and dose of the antihypertensive agent [125]. The choice of agent must also take into consideration the severity of the hypertension and the urgency of blood pressure control. In addition, several other considerations may influence the choice of antihypertensive therapy. For patients who develop hypertension while receiving treatment with an antiangiogenic agent, blood pressure should be monitored actively during the therapy. More frequent measurements in the first several weeks of treatment are prerequisite. Only this group of patients have recommendations in 2016 ESC Position Paper on cancer treatments and cardiovascular toxicity, which was published under the auspices of the ESC Committee for Practice Guidelines [126]. In these patients, treatment with angiotensin system inhibitors (ASIs; eg, angiotensin converting enzyme inhibitors [ACEIs], angiotensin receptor blockers [ARBs]) may be preferred over other drugs. In subjects treated with VEGF inhibitors, when diarrhea is observed as a side effect, diuretics should not be given as first line hypotensive therapy. As sorafenib and sunitinib undergo partial metabolism via cytochrome P450, a system inhibited by some antihypertensive agents (eg, verapamil, diltiazem) [54], therefore, these agents should probably be avoided in patients who develop hypertension while receiving sorafenib or sunitinib. In patients treated with cardiotoxic chemotherapy, who are considered Stage A Heart Failure (HF) [127], the most effective agents are generally considered to be those that are also effective at preventing adverse cardiac remodeling, including ACEis, beta-blockers, or ARBs. ACEi and beta-blockers are preferred as hypotensives in subjects with HF, left ventricular dysfunction or at risk of HF. Valuable option could be nebivolol (beta1-blocker) due to its properties affecting cell NO signaling or carvedilol with its vasodilation properties. Phosphodiesterase 5 inhibitors such as sildenafil or tadalafil may be taken into account, however, data on the efficacy are limited in this setting. Diuretics may lead to electrolyte disturbances and consequent QT prolongation, thus should be used cautiously. Beta-blockers and ACE inhibitors may be administered together with trastuzumab as prophylactic agents in patients with breast cancer as tolerated. Cardiac function could recover early after the injury caused by cancer treatment. It appears that late complications for ischemic heart disease, hypertension and rhythm disturbances are underappreciated. However, therapy of severe cardiotoxicity related to cancer treatment follow paradigms, which exist for chronic HF and ischemic heart disease, however outcomes for subjects with malignancy are different from those outcomes reported for general population. In a case of severe hypertension, close monitoring and adherence to therapy is strongly recommended. Ambulatory blood pressure monitoring is to be considered in certain cases, especially treated with VEGFR inhibitors, particularly in the first weeks of the therapy. It is also of utmost importance to assess the efficacy and tolerability of hypotensive treatment. Resistant hypertension, which is defined as inability to reach target blood pressure despite three hypotensive drugs including diuretic in adequate doses require consult of cardiooncology or hypertension specialist in order to minimize the intervals in the VEGF inhibitors therapy. Although VEGF signaling pathway inhibitors (anti-angiogenic therapy) are strongly associated with hypertension and cardiovascular dysfunction during therapy [128–131], the long-term effects of these agents remain undefined. Patients who developed hypertension during anti-VEGR therapy should continue with this antineoplastic treatment due to suggested potential clinical benefits and start antihypertensive drugs to control blood pressure. Thus, The Investigational Drug Steering Committee of the National Cancer Institute formed a Cardiovascular Toxicities Panel, joining members of its Angiogenesis Task Force with experts in the treatment of hypertension in oncology patients, developed consensus recommendations for risk assessment, monitoring, and safe administration of angiogenesis inhibitors [105]. They recommend to perform a pretreatment evaluation and screening, with formal risk assessment for potential cardiovascular complications to identify and treat preexisting hypertension before using these agents. They also stress that is it is crucial to adequately control pain and stress. Other drugs that may influence blood pressure control such as glucocorticosteroids, nonsteroidal antiinflammatory drugs, erythropoietin stimulating agents should be also taken into account. Judicious blood pressure control and aggressive cardiovascular risk factor modification are important guiding principles. Antihypertensive therapy is crucial to manage hypertension during certain chemotherapy and those agents known to prevent HF are preferred. In addition, it has been also suggested that in patients with high risk of cardiotoxicity, aggressive treatment of preexisting hypertension should be employed as suggested by Carver *et al.* [132].

### Possible influence of some cardiovascular drugs on cancer treatment

Cancer is associated with increased risk of thromboembolic events. Aki *et al.* [133] in the Cochrane Database systemic reviews concluded that heparin appeared to have no effect on mortality in cancer patients. Anticoagulation reduced the incidence of symptomatic thromboembolic events together with increased incidence of bleeding. In the recent study by Uppuluri *et al.* [134], direct acting anticoagulants-DOACs were reported to be as safe and effective as low molecular weight heparins-LMWH in cancer subjects with thromboembolism. Similar data were reported by others [135–137]. Melloni *et al.* [138]. in the ARISTOTLE trial (Apixaban for Reduction in Stroke and Other Thromboembolic Events in Atrial Fibrillation) assessed the effects and safety of apixaban vs warfarin in subjects with atrial fibrillation and history of malignancy. In this study, prior cancer was not associated with higher stroke risk. In addition, in patients with history of malignancy apixaban appeared superior to warfarin in regard to the efficacy and safety. Maraveyos *et al.* [139] showed in oncology patients an encouraging data on the use of DOACs in cancer-associated thrombosis, but they stated that LMWH remained a standard anticoagulation in this set of patients. Haaland *et al.* [140] studied the association between use of warfarin and cancer in subgroup of patients from Norwegian National Registry coupled with the Norwegian Prescription Database and the Cancer Registry of Norway. They found that warfarin users had a significantly lower age- and sex-adjusted IRR-incidence rate ratio in all cancer sites and in prevalent organ-specific sites such as lung, prostate and breast when compared to nonusers. They concluded that warfarin use in patients over 50 years of age may yield additional benefit when there was a necessity of anticoagulation. However, we should bear in mind that dose of all anticoagulant, in particular of DOACs should be adjusted to kidney function [141]. Some DOACs are to be avoided in end-stage kidney disease, in this setting LMWH or warfarin should be considered. It has been shown that low dose acetylsalicylic acid –ASA may have a potentially beneficial effect in cardiovascular disease primary prevention in cancer patients in term of mortality [142]. However, possible side effects such as bleeding should be considered before introduction of ASA as a chemopreventive modality [143]. In 2016, the US Preventive Services Task Force recommended to start ASA as cardiovascular disease and colorectal cancer primary prevention among subjects 50–59 years of age being at increased risk for cardiovascular disease. [142]. In addition, Matsuo *et al.* [144] reported that low-dose aspirin in endometrial cancer contribute to the improved survival outcomes, in particular, in young, obese, with low-grade disease, with postoperative radiotherapy.

Metformin is widely used to treat type 2 diabetes. However, metformin may act also on the different pathway i.e. may activate of LKB1 (liver kinase B1)/AMPK (5’AMP-activated protein kinase) pathway, inhibit of cell division and/or promotion of apoptosis, promote of autophagy, down-regulate circulating insulin, activate the immune system and inhibit mTOR dependent pathways and thereby exert antineoplastic properties [145–147] [145–147]. In several studies, it has been shown that metformin lowers cancer mortality when compared with either nonusers or use of other hypoglycemic drugs [148–152]. In cervical cancer metformin use was associated with improved PSF, but not in OS in diabetic patients with this cancer [153]. In addition, metformin use was reported to be associated with a lower risk of developing head and neck cancer in diabetic patients [154] but not of RCC [155].

## SUMMARY

Cardiovascular status is prerequisite to introduce certain anticancer therapy and on the other hand, effects of chemotherapy and targeted drug treatment are extremely important to cancer survivors in regard to the overall health and quality of life. Hypertension is a common comorbidity in patients with malignancy, especially in elderly population. Hypertension as a comorbidity in cancer patients was not thoroughly investigated. Some cancers may cause secondary hypertension mainly due to loss of kidney function after nephrectomy. Both chemotherapy and targeted therapy may be associated with development or worsening of preexisting hypertension. Several drugs such as cisplatin derivatives, mTOR inhibitors, anthracyclines, alkylating agents may cause hypertension. VEGFR inhibitors are the most common targeted drugs associated with hypertension as a side effect of the therapy. However, data concerning antitumor activity and hypertension are inconsistent. Problem of hypertension induced by anticancer treatment is vital because new agents, especially targeting VEGF pathway, are used frequently to treat common malignancies. Additionally, cancer patients are not always in a comprehensive cancer centers with specialists managing both the malignancy and other adverse events resulting either from the cancer or its therapy. In particular, in rural or small urban areas, oncological patients are often treated or co-treated by a primary care physician with help from a medical oncologist that may be quite far away. As shown previously, Charlson Comorbidity Index with hypertension included significantly predicted survival from all causes (HR = 1.32, 95%CI 1.18–1.49), competing causes (HR = 1.52, 95%CI 1.32–1.76) and breast cancer specific causes (HR = 1.18, 95%CI 1.03–1.35) [156]. The authors concluded that as hypertension has prognostic significance it may be necessary to introduce hypertension-augmented Charlson Comorbidity Index and include other comorbidities to this index. There are no substantial long-term data is available and guidelines for a special therapeutic strategy in cancer patients. In regard, to the management of hypertension in malignancy, meticulous attention should be paid to pretreatment screening for risk factors. In daily clinical practice, the initial treatment usually include drug affecting renin-angiotensin aldosterone system i.e. ACE inhibitor or ARB, or a long-acting calcium channel blocker-CCB most often amlodipine. The most common dual combination regimen to reach the target blood pressure consist of ACE inhibitor or ARB and either a long-acting-CCB or thiazide diuretic. In three drug therapy the preferred regimen consists of ACE inhibitor or ARB with a long-acting CCB and a diuretic. In subjects with at least stage 4 of CKD i.e. an estimated glomerular filtration-eGFR rate of less than 30 mL/min per 1.73 m^2^, a loop diuretic, such as furosemide or torsemide, is added for effective volume control. In the recent paper from Journal of American College of Cardiology (14th Nov) only a small section with very limited data is devoted to the hypertension [157]. A recently published survey revealed that almost all medical oncologists administered cardiotoxic treatments, including anthracyclines (83%), trastuzumab (51%) and other antiangiogenic drugs (64%) [158]. Only 35% of oncologists managed cardiotoxicity on the basis of the guidelines from expert oncology societies, whereas recommendations from expert cardiology societies was virtually not known. In addition, the treatment of hypertension was not consistent. The authors concluded that oncology practices are disparate in the area of cardiotoxicity.

Therefore, wrapping up, as the aging population increases both the risk of hypertension and cancer, thus urgent need for cooperation between oncologists, cardiologists, nephrologists or hypertension specialists to effectively manage cancer patients with hypertension as a comorbidity or a complication of the therapy. Researchers should at first assess the epidemiology of hypertension in cancer patients, then look at the possible influence of history of hypertension on development of serious side effects and outcomes, and whether appropriate and timely therapy affect survival and quality of life.
